# Reproductive justice and support for young fathers

**DOI:** 10.1002/imhj.21806

**Published:** 2019-07-19

**Authors:** Andre Dukes, Glen Palm

**Affiliations:** ^1^ Northside Achievement Zone Minneapolis Minnesota; ^2^ Department of Child and Family Studies St. Cloud State University St. Cloud Minnesota

**Keywords:** child development, fatherhood programs, reproductive justice, untapped resource, young fathers, papás jóvenes, justicia reproductiva, programas para la paternidad, desarrollo del niño, recurso sin explorar, Jeunes pères, Justice reproductive, Programmes de paternité, Développement de l'enfant, Resource inexploitée, Junge Väter, Reproduktive Gerechtigkeit, Vaterschaftsprogramme, Kindesentwicklung, ungenutzte Ressourcen, 若年の父親, 生殖の権利, 父性プログラム, 子どもの発達, 未開発の資源, 年輕父親, 生殖公義, 父權計劃, 兒童發展, 未開發資源, الآباء الشباب, العدالة الإنجابية, برامج الأبوة, نمو الطفل, الموارد غير المستغلة

## Abstract

The current article addresses the interests and contributions of fathers to child development and well‐being within a reproductive and social justice framework. We present an overview of research on the role of fathers in the lives of children from the prenatal period through early childhood, with an emphasis on fathers as partners and caregivers in promoting the reproductive health and safety of women and the healthy development of young children. We explore especially the challenges of young, at‐risk fathers as well as system and practice opportunities that support their contributions as partners and parents. Our goal of the article is to extend the discourse on reproductive and social justice to include the shared responsibility of all parents and facilitate circumstances whereby children experience the support needed to become nurturing caregivers for the next generation.

## RESUMEN

El presente artículo se refiere a los intereses y contribuciones de los papás al desarrollo y bienestar del niño dentro de un marco de justicia reproductiva y social. Presentamos una revisión general de la investigación sobre el papel de los papás en la vida de niños desde el período prenatal y a través de la primera infancia con énfasis en los papás como compañeros y cuidadores en el proceso de promover la salud y seguridad reproductiva de las mujeres y el desarrollo saludable de los pequeños niños. Exploramos especialmente los retos de papás jóvenes bajo condiciones de riesgo, así como oportunidades sistemáticas y prácticas que apoyan sus contribuciones como compañeros y progenitores. La meta del estudio es extender el tema de la justicia reproductiva y social para incluir la responsabilidad compartida de todos los padres y facilitar las circunstancias por medio de las cuales los niños experimentan el apoyo necesario para convertirse en fomentadores de una crianza propicia para la próxima generación.

## RÉSUMÉ

Cet article porte sur les intérêts et les contributions des pères au développement de l'enfant et à son bien‐être dans le contexte de justice reproductive et de justice sociale. Nous présentons un survol des recherches sur le rôle des pères dans les vies des enfants de la période prénatale à la petite enfance avec l'accent placé sur les pères en tant que partenaires et modes de soin promouvant la santé reproductive et la sécurité des femmes et le développement sain des jeunes enfants. Nous explorons spécialement les défis auxquels font face les jeunes pères à risque ainsi que les systèmes et les opportunités de pratiques qui soutiennent ces contributions en tant que partenaires et parents. Le but de cet article est d’élargir le discours sur la justice reproductive et la justice sociale afin d'inclure la responsibilité partagée de tous les parents et de facilier les circomstances dans lesquelles les enfants font l'expérience du soutien nécessaire afin de devenir à leur tout des parents protecteurs et chaleureux pour la prochaine génération.

## ZUSAMMENFASSUNG

Der aktuelle Artikel befasst sich mit den Interessen und Beiträgen von Vätern zur Entwicklung und zum Wohlbefinden ihrer Kinder im Rahmen der reproduktiven und sozialen Gerechtigkeit. Wir geben einen Überblick über die Forschung zur Rolle der Väter im Leben ihrer Kinder von der Pränatalzeit bis zur frühen Kindheit, wobei der Schwerpunkt auf den Vätern als Partner und Bezugspersonen bei der Förderung der reproduktiven Gesundheit und Sicherheit von Frauen und der gesunden Entwicklung von Kleinkindern liegt. Wir untersuchen insbesondere die Herausforderungen junger, gefährdeter Väter sowie System‐ und Übungsmöglichkeiten, die ihre Beiträge als Partner und Eltern fördern. Ziel des Artikels ist es, den Diskurs über reproduktive und soziale Gerechtigkeit so zu erweitern, dass er die gemeinsame Verantwortung aller Eltern einbezieht und Umstände begünstigt, unter denen Kinder die notwendige Unterstützung erfahren, um fürsorgliche Bezugspersonen für die nächste Generation zu werden.

## 抄録

生殖の権利と若年の父親への支援

この論文は、生殖や社会的権利の枠組みにおける子どもの発達や福祉に父親が関心を持つこと、貢献することについて述べた。われわれは、周産期から乳幼児期にある子どもの生活において、女性の生殖に関連する健康と安全や、幼児の健康的な発達を促進する際のパートナーやケアをする人としての父親に強調点を置いた、父親役割に関する研究を概観した。われわれは、とくに若年で虐待のリスクのある父親の困難のみならず、パートナーや親として彼らが貢献することを支援するシステムや取り組むための機会について調査した。論文の目的は、子どもたちが次世代の養育者になるために必要な支援を経験していけるよう、すべての親が責任を分かち合うことを含む、生殖や社会的権利に関する議論を広げ、そのような状況を促していくことである。

## 摘要

年輕父親的生殖公義和支持

本文涉及父親在生殖和社會公義框架內對兒童發展和福祉的興趣和貢獻。我們概述父親在產前期到幼兒期如何影響兒童生活的研究, 重點是父親作為合作夥伴和照顧者, 以促進婦女的生殖健康和安全以及幼兒的健康成長。我們特別探討年輕丶有風險父親的挑戰, 以及支持他們作為合作夥伴和育兒者的系統和實踐機會。該文件的目標是擴大關於生殖和社會公義的討論, 包括所有父母的共同責任, 並促進兒童獲得為下一代培養照顧者所需的支持。

## ملخص

العدالة الإنجابية ودعم الآباء الصغار

تتناول هذه المقالة مصالح ومساهمات الآباء في نمو الطفل ورفاهه في إطار العدالة الإنجابية والاجتماعية. نقدم لمحة عامة عن دور الآباء في حياة الأطفال من فترة ما قبل الولادة وحتى مرحلة الطفولة المبكرة مع التركيز على الآباء كشركاء ومقدمي رعاية في تعزيز الصحة الإنجابية وسلامة المرأة لصحة نمو الأطفال الصغار. ونستكشف على وجه الخصوص التحديات التي يواجهها الآباء الشباب المعرضون للخطر، فضلا عن فرص النظام والممارسة التي تدعم مساهماتهم كشركاء وآباء. والهدف من الورقة هو توسيع نطاق الخطاب المتعلق بالعدالة الإنجابية والاجتماعية ليشمل المسؤولية المشتركة لجميع الوالدين وتيسير الظروف التي يتعرض فيها الأطفال للدعم اللازم ليصبحوا مقدمي الرعاية للجيل التالي

It is impossible to separate involved and equitable fatherhood from reproductive justice and broader social justice. (Heilman, Cole, Matos, Hassink, Mincy, & Barker, 2016, p. 31)

## 1 INTRODUCTION

Reproductive justice, as a dimension of a social justice movement, is rooted in the principle that individuals, families, and communities have the resources and power to make sustainable decisions about their lives and the lives of their children and to parent their children in safe and healthy environments (Heilman et al., 2016; St. John, Thomas, Norona, and the Irving Harris Foundation Professional Development Network, 2012). Often overlooked in the goals of reproductive and social justice work are the reproductive and parenting interests of men and fathers. In moving toward an ethic of gender equity, there is need for a clear vision of men (including men who are racial and ethnic minority fathers, immigrants, incarcerated, young, gay) as equal partners in reproductive decision‐making and the physical and emotional care of dependent family members (Levtov, van der Gaag, Green, Kaufman, & Barker, 2015). As Levtov et al. (2015) envisioned:

Fathers can help break the cycle of violence and discrimination against women by modeling non‐violent behaviors and instilling values of equality, respect for diversity, empathy, and human rights for the next generation. They can act confidently as caregivers to both children and aging parents, and can make an equal investment in domestic duties and the provision of household necessities. When men take on more care responsibilities, it empowers women to find paid work outside the home, to improve their health and education, and to take on leadership roles. This is good for everyone: women and girls, men and boys. (p. 6)

From this gender‐equity lens, in the current article we provide a brief overview of research on the role of fathers in the prenatal to early childhood period as partners, protectors, and caregivers. From a family systems perspective, we emphasize the importance of building capacity in especially young, unmarried fathers in parenting roles while safeguarding the safety, reproductive health, and rights of women. The overall goal of this review is to deepen the discourse on reproductive and social justice to include a shared responsibility of women and men for reproductive concerns and child‐rearing as a foundation for diverse family systems and healthy child development.

## 2 FATHERS AS CAREGIVERS: SOCIAL AND ECONOMIC CONTEXT

Parental roles and family structures have evolved with changes in economic and social conditions that impact fathers as caregivers (McLanahan, 2011; Sawhill, 2014), especially young unmarried fathers (Edin & Nelson, 2013). Changes in the social context for families can be traced back to the sexual revolution of the 1960s, with an increase in sexual activity prior to marriage for both men and women (Sawhill, 2014). There also has been a growing disconnect between marriage and parenthood so that by 2015, 40% of children were born to unmarried parents (Martin, Hamilton, Osterman, Driscoll, & Matthews, 2017). Multipartner fertility, parents who have children with more than one partner, also has increased and is higher among men than women among cohabitating families (38.8% for women, 64.2% for men) in the Fragile Families sample (McLanahan, 2011). Families where multipartner fertility exists reflect increased complexity and instability (Sawhill, 2014). These social factors interface and collide with the expectation that men should be more involved in their role as fathers (Machin, 2015; Primus, 2017).

The relevant economic factors that influence the role and involvement of fathers include the changing economy from an industrial to an information‐ and service‐based economy (Sawhill, 2014). Changes in men's education levels and the loss of high‐paying jobs for men with limited education has led to a high rate of unemployment for men (22% of men ages 20–64) (Eberstadt, 2016). Young, low‐educated minority men have even higher rates of unemployment (30% for young Black men ages 16–24) (Institute for Research on Poverty, 2014). The lack of employment for men participating in Responsible Fatherhood programs was a major challenge for 65% of the men interviewed (Fagan & Kaufman, 2015). A related social change with economic implications has been the increase in incarceration rates that have impacted families of color in an unequal manner (Alexander, 2012). This confluence of social and economic change factors has created new challenges for father involvement at a time when developmental research and family systems perspectives have informed our understanding of the changing contributions of fathers in child development (Black, Dubowitz, & Starr, 1999; Cabrera & Tamim‐LeMonda, 2013). Increasingly, from conception, the responsibility for the health and well‐being of both mother and child is shared by the mother, the father, and other caregivers (Cabrera & Tami‐LeMonda, 2013; Lamb, 2010; Parke, 2013a). Decades of research has suggested that in terms of dual responsibility, both mothers and fathers are capable of providing basic caregiving that infants and young children need for survival and healthy development (Lamb, 2010). There is also evidence that multiple caregivers make independent contributions to children's social, emotional, and cognitive development and that mothers and fathers differ in type, degree, and quality of parenting interactions and experiences related to young children (Parke, 2013b).

Although father presence and involvement are multidimensional constructs with definitions that vary historically (Palkovitz, 1997; Parke, 2013a), overall, father presence has been demonstrated to be a protective factor for children in adverse environments (Fitzgerald & Bocknek, 2013; Martinez, DeGarmo, & Eddy, 2004; McHale, 2007), and direct, consistent involvement of fathers in children's lives has been associated with the positive socioemotional and cognitive development of children (Parke, 2013a, 2013b). While biological fathers are more likely to be involved directly with their children, residential status and the broader family context play significant roles in level of paternal involvement, especially as new family structures evolve (Edin & Nelson, 2013; McLanahan, 2011). Father figure absence (and inconsistent presence) has been linked to poor educational, behavioral, and developmental outcomes for children, with both direct and indirect effects influenced by environmental context (e.g., partner relationship quality, life stress, social support, employment, and educational opportunities; Cabrera & Tamis‐LeMonda, 2013; Fitzgerald & Bocknek, 2013; Lamb, 2010; Parke, 2013a; Pleck, 2010; Sroufe, Egeland, Carlson, & Collins, 2005; Tamis‐LeMonda, Niwa, Kahana‐Kalman, & Yoshikawa, 2008). In general, environmental factors are thought to interact with biological mechanisms to influence the development and maintenance of paternal (as well as maternal) engagement and caregiving behavior (Storey & Walsh, 2013).

## 3 FATHERS AS CAREGIVERS: BIOLOGICAL MECHANISMS

In mammalian species, paternal care occurs in less than 10% of species whereas maternal care is central in all (Kleiman & Malcolm, 1981). From an evolutionary perspective, it is hypothesized that paternal care, or two‐parent care, may have evolved as a result of difficult environmental conditions, with multiple benefits for offspring such as increased growth rates and survival of the young (e.g., Alvergne, Faurie, & Raymond, 2009; Hurtado & Hill, 1992, Waynforth, 2013).

Relative to maternal behavior, the biological basis of paternal care has been less well‐studied, in part due to the low frequency and high variability of paternal involvement (Storey & Walsh, 2013). However, recent findings related to hormonal variation in naturally paternal species have resulted in renewed study of hormonal (e.g., prolactin, steroid hormones, oxytocin, glucocorticoids) and neurobiological mechanisms in human paternal care (e.g., Gray & Anderson, 2010; Gray, Parkin, & Samms‐Vaughn, 2007; Storey & Walsh, 2013). For example, lower levels of testosterone have been found in married versus single men (Burnham et al., 2003), in fathers during their partners’ late pregnancy and early postpartum periods (Perini, Ditzen, Hengartner, & Ehlert, 2012, Storey, Walsh, Quinton, & Wynne‐Edwards, 2000), and in men who provide more paternal care versus those who provide less care or men without children (Gettler, McDade, Feranil, & Kuzawaa, 2011). Moderate (regulated vs. high or low) levels of testosterone have also been associated with better infant care, suggesting the hormonal reduction may be a consequence rather than a cause in developing fatherhood (Rilling, 2013). Lower testosterone levels (and associated behavioral levels of aggression) in men have also been associated with affiliative interactions with a romantic partner (Burnham et al., 2003). Together, these and related findings have suggested that adaptive biological changes may be associated with affective communications between father and child as well as between adult caregivers.

On the continuum of mammalian paternal (vs. maternal) care, human paternal responsiveness is not likely to be biologically driven but to require socialization through nurturance experienced early in development and exposure in adulthood enhanced by cues from mates and infants (Kim et al., 2014; Storey & Walsh, 2013). Consistent with this idea, early experiences in father‐present versus father‐absent homes have been found to influence whether boys grow up to be paternally responsive and capable of committed relationships (Belsky, Steinberg, & Draper, 1991), and early exposure to children within the family yield biological and behavioral changes related to responsivity to subsequent infant cues (Delahunty, McKay, Noseworthy, & Storey, 2007). Further, differences in male and female neurobiology and socialization are thought to contribute independently to offspring development and well‐being. Ingalhalikar et al. (2014) suggested that sex differences in brain structure, connectivity, and hemispheric communication dominance distinguish male and female socioemotional developmental trajectories early in life. Genetically and epigenetically shaped by social and physical environments, adult male and female neurobiological differences may represent “adaptive complementarity for optimal human function” (Schore, 2017, p. 42).

## 4 FATHER CONTRIBUTIONS TO CHILD DEVELOPMENT

Father (as mother) involvement with children typically emerges prenatally with the anticipation of a future caregiving role. Many fathers report feelings of connectedness to a future child, which may prepare men psychologically for postnatal parenthood adjustment (Hjelmstedt & Collins, 2008). Moreover, prenatal involvement has been shown to be a strong predictor of later father involvement (Cabrera, Fagan, & Farrie, 2008). Father support of the pregnancy has also been related to positive effects on maternal experience, including prenatal care usage, abstinence from alcohol and smoking, experience in labor and delivery, and postnatal health (Redshaw & Henderson, 2013) as well as to reduction in low birth weight and small‐for‐gestational‐age infants (e.g., Alio, Kornosky, Mbah, Marty, & Salihu, 2010) and to an increase in postnatal breastfeeding rates (Maycock et al., 2013). However, men adapt to the transition to fatherhood in diverse ways. Research has suggested that men who feel “unready” for fatherhood tend to find the transition challenging and are less committed and involved as parents. Father age, parity, ethnicity, and social deprivation have been found to shape fathers’ reactions to and degree of involvement with pregnancy, perinatal, and birth experiences as well as postnatal care (e.g., Redshaw & Henderson, 2013).

In the newborn period, fathers have been found to interact tenderly (as do new mothers) and to learn quickly about the uniqueness of their own newborn children, adjusting their speech and singing patterns (more slowly and with a high pitch, using shorter phrases, imitation, and repetition) in response to infant cues (Lamb & Lewis, 2013). Variations in father involvement, especially in infant care, have been related to factors such as prior parenting opportunities, personality characteristics and self‐perceptions, childbirth circumstances (premature birth), and child gender (Lamb & Lewis, 2013), with greater involvement associated with greater sensitivity across early childhood (Lamb, Chuang, & Hwang, 2004).

Caregiver sensitivity and responsiveness is central to the development of healthy infant–caregiver attachment relationships. Prompt, appropriate, and reliable care in response to infant signals support the development of secure attachments and confidence in the availability of adult care (e.g., DeWolff & van IJzendoorn, 1997; Sroufe et al., 2005; van IJzendoorn & DeWolff, 1997). Research has demonstrated that infants form attachments to both parents (and other caregivers) provided that the infant has sufficient and stable access to adult interaction (Lamb, 1977a, b; Steele, Steele, & Fonagy, 1996). Approximately 65% of attachments to both mothers and fathers are rated as secure (e.g., Ahnert, Pinquart, & Lamb, 2006). However, infant relationships with fathers and mothers may derive from different social experiences, with mothers typically providing security related to child emotional distress and fathers promoting security through sensitive support in exploration and play especially in the toddler period (e.g., Grossmann, Grossmann, Kindler, & Zimmermann, 2008). Consistent with these findings, when both parents are present, distressed 12‐ to 18‐month‐olds tend to exhibit a preference for maternal care whereas 18‐ to 21‐month‐olds show no comparable preference (see review by Lamb & Lewis, 2010). Especially between 10 and 20 months of age, mothers appear to be reliable sources of comfort and security whereas fathers may be preferred partners for playful interaction. Regardless of these differences, infants experience and are responsive to information and interaction from both parents (Lamb & Lewis, 2013; Parke, 2013b).

Through the toddler and the preschool periods, fathers and mothers appear to continue to engage in different types of interactions with their children, with fathers engaged in more physical play, characterized by arousal, excitement, and an unpredictable pace of interaction and mothers engaged in more modulated, conventional, and toy‐ or verbally mediated activities (Borke, Lamm, Eickhorst, & Keller, 2007, Lamb & Lewis, 2013; Parke, 2013b). Across development, paternal interactive styles have been linked with positive child outcomes, including reduced aggression, improved peer relationships, and the capacity to cope with novelty and challenge (Parke, 2013b), even controlling for contributions from maternal interactions (Leidy et al., 2011). Links between interactions of fathers (and mothers) with their young children and socioemotional functioning are thought to be derived from the internalization of the relational experience of emotional regulation (Parke, 2013a; Sroufe et al., 2005). Despite these documented historical differences, parental interaction styles vary across cultures, and defined gender‐based differences may diminish with trends toward increasing father involvement in core caregiving activities (for a review of father–child involvement and child outcomes, see Lamb & Lewis, 2010; Pruett, 2000).

From a family system perspective, fathers influence young children directly through interaction and indirectly through relationships with mothers (just as mothers influence paternal behavior, and children influence parents; Parke, 2013a; Sameroff, 2009). The quality of marital (or partner) relationships has been demonstrated to be a critical indicator of parental interaction with children from infancy forward (Gable, Crnic, & Belsky, 1994). For example, marital and nonresidential parent–infant relationship quality have been associated with sensitive caregiving behavior of both mothers and fathers as well as positive child outcomes (e.g., Clarke‐Stewart & Brentano, 2006; Goldberg & Easterbrooks, 1984). Adult relationship quality appears to be an especially important predictor of father–child relationships, influencing father attitudes, sensitivity, warmth, and caregiving behavior toward their infants (Cox, Owen, Lewis, & Henderson, 1989; Heinicke & Guthrie, 1992). Differences in involvement and quality of parent–child interaction also reflect maternal attitudes and behaviors regarding the fathering role (Fagan & Barnett, 2003; Wood & Repetti, 2004). In general, parenting behavior and the quality of parent–child relationships are inseparable from the dynamic relationships between adults and contextual family circumstances (e.g., employment, work demands, parental depression, availability of extended family; Belsky, 1996; Goodman, Crouter, Lanza, & Cox, 2008; Hetherington & Kelly, 2002; Roggman, Bradley, & Raikes, 2013; Sroufe et al., 2005). Although father–child relationships have been found to be especially influenced by contextual factors (e.g., Belsky, 1996), recent research has focused primarily on married fathers in families or families with absent fathers. With increasingly diverse patterns of family organization (Parke, 2013a; Sawhill, 2014), there is a need for further exploration of contextual factors influencing father caregiving behavior.

## 5 EXPERIENCES OF YOUNG FATHERS

Of particular concern are the experiences of young fathers (ages 18–25) growing up in poverty or at‐ risk environments for whom becoming a parent is especially challenging (Berger & Langton, 2011; Primus, 2017). Young fathers are often unmarried and lack support for both adult partnership as well as parenthood (McLanahan, 2011; Primus, 2017). Although statistics regarding marital or partner status are often incomplete (Martin et al., 2015; Minnesota Fathers and Families Network, 2007), the number of nonmarital births has risen for 30 years, leveling off at approximately 40% for the general population of mothers (Child Trends, 2015). Nonmarital births vary by race from a low of 17% for Asian and Pacific Islanders, 29% for Whites, 53% for Hispanics, 66% for American Indians, and 72% for Blacks (Child Trends, 2015). Available data have suggested that 88% of births to men under 20 years of age are nonmarital, and 53.9% of births to men 20 to 24 years are nonmarital versus 19% to fathers 25 to 44 (Martinez, 2015). Young fathers of diverse backgrounds are often not counted in birth records (Martin et al., 2015); they become invisible and generally have limited influence on decisions related to reproductive health and child well‐being (Carlson & McLanahan, 2010).

Young fathers also face a range of challenges to involvement in their children's lives. These may include educational, economic, and legal issues as well as negative social experiences (e.g., childhood trauma) and messages (e.g., regarding the capacity of young men to be caring fathers and partners) (Fagan & Kaufman, 2015). For example, young fathers complete lower than average levels of education and experience higher rates of unemployment (Institute for Research on Poverty, 2014). Moreover, opportunities in the labor market and incarceration rates without a high‐school diploma disproportionately impact young fathers, especially young men of color (i.e., 28% of Whites, 20% of Hispanics, and 68% of Blacks can expect serve at least 1 year in prison by age 30) (Institute for Research on Poverty, 2014). Efforts to disrupt this “pipeline to prison” and its negative impact on very young children are critical especially in African American communities (see Children's Defense Fund, 2007). Additional challenges include multipartner fertility, further limiting resources available for young men to invest in their children. Almost 33% of young unmarried fathers and 47% of young Black fathers have children with multiple partners (Institute for Research on Poverty, 2014). Limited educational, employment, and social pathways perpetuate a cycle of poverty for many young fathers (Edin & Nelson, 2013). For other fathers, immigrant status poses a constant threat of deportation, affecting their ability to balance cultural norms (e.g., role of provider) with limited or unstable employment opportunities (Suarez‐Orozco & Suro, 2015). While the reproductive justice movement has liberated many men from heteronormative ideas of fatherhood, there remain complex barriers for young LGBT men who wish to become a parent (e.g., shaming regarding adoption of traditional views of the family unit; Mallon, Scourfield, & Harvey, 2004). Together, this array of findings has demonstrated the marginalization of male minority groups under age 25 who face multiple diverse barriers to reproduction and parenthood. Age, race, education, sexual orientation, rates of incarceration, and complex family systems all intersect to compound disadvantages for young unmarried fathers, especially young Black fathers, and their children.

Findings from a national study of fathers in “fragile families” (Carlson & McLanahan, 2010) have illustrated the circumstances of many young fathers during family formation. In this study, at the birth of the child, 82% of the young unmarried fathers were found to be romantically involved with their female partners, with 50% cohabitating and 32% visiting. Only 10% of partners reported no contact with the father at the time of birth. These relationship patterns varied by race, with White and Hispanic fathers more likely to be cohabitating with mothers (65 & 60%, respectively) and 40% of Black fathers cohabitating at the time of birth. These young men reported valuing marriage as “better for the child” (78%), valuing the father role, and providing early financial support at the time of birth. Five years following the birth, 20% of fathers were married; however, others had no relationship (42%) or were friends (20%). Positive father attitudes toward marriage or partnership and motivation to be involved with offspring were frequently thwarted by challenges ranging from employment to complex family structures and social relationships (Bronte‐Tinkew, Horowitz, & Scott, 2009; Grych & Clark, 1999).

Healthcare systems also pose unique challenges for young, unmarried fathers. Education and support services for young fathers are not well‐integrated into existing healthcare systems and funding sources that serve families with young children (Draper & Ives, 2013; Yogman & Garfield, 2016). Beginning in the prenatal period, men often encounter healthcare and insurance policies and practices focused exclusively on mothers and birth outcomes, with minimal attention to the interests and needs of young fathers. Social and mental health services for young men are frequently directed toward intervention/treatment after problems have emerged, with preventive and early intervention fatherhood programs restricted to short‐term or pilot projects that serve a small number of fathers (Klempin & Mincy, 2012). For many at‐risk young fathers, social and educational services first become available when they enter the criminal justice system (Palm, 2003).

Young men also face less visible challenges to parental involvement. Young unmarried fathers often bring histories of trauma and stress, may lack role models of positive father involvement and coparenting relationships, and may live in communities where poverty and violence both limit opportunities and normalize low expectations (Cooper, 2015). Negative social stereotypes include characterizations of young fathers as nonessential or easily replaced, disinterested and uninvolved, irresponsible with respect to child support, and expected consequences of intergenerational cycles of poor parenting (Carlson & McLanahan, 2010; Tamis‐LeMonda & McFadden, 2010). However, at the same time as research highlights visible and invisible contextual challenges to young father involvement (e.g., lack of residential status and contact with the child, maternal gate‐keeping, complex family systems; Bronte‐Tinkew et al., 2009; Carlson & McLanahan, 2010; Fagan & Barnett, 2003), notable improvement in father involvement is linked with support from mental health services, flexible employment (e.g., part‐time work hours), and increased education (Hofferth, Pleck, Goldscheider, Curtin, & Hrapczynski, 2013).

Appendix A provides a common narrative regarding the experience of many young fathers. Whereas poverty and new parenthood pose challenges for both men and women, young mothers frequently experience educational, employment, and caregiving support. In contrast, efforts to engage fathers (with the exception of collecting child support) are often limited by system policies and practices and the lack of social encouragement. Related or in addition, young fathers often drift into new romantic relationships with responsibilities for additional children and few options to make their family lives work (Carlson & McLanahan, 2010; Heilman et al., 2016, Sawhill, 2014). This example demonstrates the potential negative influence of inequitable service opportunities for young fathers and the cascading negative effects for mother and child.

## 6 POLICY AND PRACTICE IMPLICATIONS

Decades of research focusing on the experiences and challenges of fathers have spawned recommendations for policy and system level change as well as community and program level practices that address the needs of young, unmarried fathers (e.g., Bronte‐Tinkew, Burkhauser, & Metz, 2008; Cowan, Cowan, Pruett, Pruett, & Wong, 2009; Deslausiers, Devault, Groulx, & Sevigny, 2012; May & Fletcher, 2013; McHale & Phares, 2015; Panter‐Brick et al., 2014; Primus, 2017; St. John et al., 2012).

### 6.1 Policy‐ and system‐level changes

Policy‐ and system‐level change recommendations emphasize (a) early intervention, beginning as early as possible with expectant fathers; (b) adoption of a diverse family systems perspective; (c) creation of a continuum of parent education and support services that include fathers; and (d) collaboration among service sectors. Importantly, early intervention efforts take advantage of the openness and motivation of young men during the transition to fatherhood to build paternal capacity and participation in child‐rearing issues during a vulnerable time for both mother and developing child (e.g., Bond, 2010; Burgess, 2008; Center for the Developing Child, 2016; Draper & Ives, 2013; Fagan, 2008; Genesoni & Tallandini, 2009; Hoffman, 2011; Panter‐Brick et al., 2014; Roggman, Boyce, Cook, Christiansen, & Jones, 2004). Prenatal and perinatal transitions offer opportunities for expectant fathers to learn about protective influences that reduce stress for both parents and improve birth outcomes (Bond, 2010; Burgess, 2008). Preparation for fatherhood and caregiving participation also provide opportunities for young men to address unresolved issues related to their own early caregiving experiences, especially concerning adversity (i.e., Skjothaung, Smith, Wentzel‐Larsen, & Moe, 2015).

Second, a diverse family systems framework, beginning during pregnancy, challenges practitioners to expand their focus to include experiences and contributions of fathers, multigenerational effects (i.e., historical trauma), multicultural perspectives, and relational approaches to service delivery to support the development of the child and family (Kim & Watamura, 2017; McHale & Phares, 2015, Parke, 2013a; St. John et al., 2012; Yogman & Garfield, 2016). Third, a continuum of services from broad educational and support services for all young parents to tailored programs that address risks may provide a “gateway” to parenthood (as well as adulthood) for young fathers while minimizing common social stigma (Fletcher, May, St. George, Stoker, & Oshan, 2014; Kotila, Snyder, & Qian, 2015; National Academy of Sciences, 2016; Primus, 2017). Finally, there is need for collaboration among the systems that serve young parents and families during pregnancy and through the transition to parenthood (Deslauriers et al., 2012; Primus, 2017). Communication among systems of healthcare, education, social welfare, and often criminal justice improves the effectiveness of service delivery and reduces duplication, contradiction, and confusion for families.

### 6.2 Community programs

Program‐level recommendations cluster around three areas of practice: (a) engagement, (b) program content, and (c) process strategies. Engagement of young fathers begins with practitioner beliefs in the value and interest of young men in the lives of their children (Primus, 2017; Sandstrom et al., 2015); in the creation of community‐based programs in familiar settings with experienced, enthusiastic professionals (Bronte‐Tinkew et al., 2008; Fagan, 2008; Fletcher et al., 2014; McHale, Salman‐Engin, & Coovert, 2015; Zaveri, Baumgartner, Dion, & Clary, 2015); and in a focus on engagement during the prenatal period (Hoffman, 2011, Sandstrom et al., 2015). Examples of programs that engage fathers during the transition to parenthood include:

- Family Foundations (Feinberg & Kan, 2008) is an eight‐session program that meets with mothers and fathers from the third trimester of pregnancy through the first months after the birth of the child. A recent follow‐up study of Family Foundations in a number of sites has indicated improved parenting and coparenting strategies and positive impacts on child adjustment after 2 years (Jones et al., 2018).
- Boot Camp for New Dads (Bishop, 2006) is a universal educational program (with links to other services) offered through healthcare providers during the transition to parenthood.
- Program P (Promundo, CulturaSalud, & REDMAS, 2013) provides practical advice for engaging fathers during prenatal visits and program “father‐friendliness” checklists for health care programs.
- Figuring it Out for the Child (McHale et al., 2015) is a six‐session coparenting intervention offered to Black mothers and fathers during the third trimester, with a booster at 1 month’ postpartum to increase awareness of the benefits of coparenting, enhance rapport and the parenting alliance, and develop communication and problem‐solving skills. Results have indicated declines in conflict and destructive interpersonal dynamics and improved rapport and problem‐solving by the couples.

These are just a few examples of programs for fathers that focus on early intervention during the prenatal period through the transition to parenthood. The need for more evaluation research has been a consistent theme for fatherhood programs and led to the establishment of the Fatherhood Research and Practice Network in 2014. The purpose of this Network is to support rigorous evaluation of fatherhood programs and to build the capacity of researchers and practitioners to collaborate and develop new tools for assessing program outcomes (Fatherhood Research and Practice Network, 2014).

Program recommendations regarding content have noted that interest and needs of expectant and new fathers vary depending upon the timing of service in relation to the child's development (e.g., Bronte‐Tinkew et al., 2009; May & Fletcher, 2013; McHale et al., 2015; Palm, 2014). However, the range of topics includes (a) prenatal processes and methods to support pregnant partners and participate in decision‐making, (b) reproductive options related to recurring pregnancies, (c) relationship and role changes in the transition to parenthood for both mothers and fathers, (d) risks to the pregnancy and infant associated with unhealthy adult behaviors, (e) risks related to partner and personal mental illness and depression and ways to seek help, (f) infant behavior and development (e.g., crying patterns, attachment relationships), and (g) caregiving skill‐building. In addition, young fathers often need to address immediate concerns related to employment, housing, education, and life stress (Deslausiers et al., 2012). Regardless of content, program information and activities must be geared to cognitive and emotional developmental capacities of individual fathers.

Overall, recommendations emphasize that program success depends on the promotion of trusting relationships between young fathers and experienced, multidisciplinary professionals through community‐based connections, case management, and mentoring (Bronte‐Tinkew et al., 2008; Deslausiers et al., 2012; Fletcher et al., 2014). Building relationships with young fathers can be facilitated by social and recreational activities, active learning approaches (e.g., building or creating something for the baby), engagement in program planning, peer and social network involvement, focus on specific skills and use of technology (video feedback to improve sensitivity to cues and synchrony; Benzies, Magill‐Evans, Harrison, Gleri, & Kimak, 2006; Feldman, 2012), flexibility in scheduling and venue (center‐ vs. home‐based), continuity in professional involvement, and collaboration with other services (e.g., education, job training, mental health, legal advice). Moreover, programs for fathers must aim to identify practical family routines and “micromoments” in which fathers can participate and practice skills (e.g., feeding, holding infants, engaging in verbal exchanges, offering children opportunities to explore their environment; Leidy, Schofield, & Parke, 2013; Tamis‐LeMonda, Kahana‐Kalman, & Yoshikawa, 2009; Tamis‐LeMonda, Baumwell, & Cabrera, 2013).

Recent studies of program‐delivery systems for young fathers have provided evidence of the effectiveness of these principles. Sandstrom et al. (2015) examined approaches to father engagement within home‐visiting services. In addition to visits to the home, programs included peer support groups, outings, and family events as supplemental activities to build social support among fathers and families. Other critical components included (a) adaptations to content and activities to meet specific interests and needs of fathers; (b) emphasis on building a trusting relationship through persistence, patience, a nonjudgmental attitude, flexibility, and advocacy; and (c) male home visitors, when possible. The study recommended collaborations with federal Responsible Fatherhood programs to address challenges in working with nonresidential fathers, teen fathers, and families where mothers limited father involvement. Fathers participating in the home‐visiting programs reported improvement in parenting skills, anger management, and partner communication.

A second study evaluated four Responsible Fatherhood programs (Zaveri et al., 2015). Directed toward young, unmarried families, these programs focused on three areas: (a) parenting/fatherhood, (b) economic stability (education and job training), and (c) healthy relationship and marriage. Key program factors identified in the study included (a) staff with similar background experiences as fathers to serve as role models and a base for building trust, (b) an assessment process to tailor program activities to fathers’ needs, and (c) collaboration with other agencies to address needs for case management, behavioral health services, and legal issues around child support. Regardless of service delivery (structured vs. open‐ended), programs have struggled to engage fathers in healthy adult relationships and marriage activities. Additional research is required to gauge the overall effectiveness of these programs.

Critical to father and diverse family system inclusive practice are reflective processes that address ethical and relational challenges (e.g., Draper & Ives, 2013; Palm 1998; Primus, 2017; Slade, 2005). One example of such practice guidelines are Core Principles for Ethical Relational Discourse (Minnesota Council on Family Relations, 2016), originally designed to address ethical dilemmas faced by parent and family educators. Appendix B provides an overview of these principles adapted to work with fathers and other family members, with the goal of honoring the needs and aspirations of all family participants (for process steps, see Minnesota Council on Family Relations, 2016). The principles reflect diversity‐informed professional tenets (e.g., St. John et al., 2012) as well as research evidence related to healthy development and effective practice with parents and families. In articulating these principles, we hope to encourage gender‐equity thinking and practice and the inclusion of fathers in reproductive and child‐rearing responsibilities while acknowledging the needs and wishes of women and children.

## 7 CONCLUSION

Decades of research in developmental science (Shonkoff, 2010; Sroufe et al., 2005) support a view of early childhood as a critical period in human development when survival and development depend on the commitment and involvement of primary caregivers. It is also a critical period for caregivers, including fathers, in the lives of children. An extensive literature supports the interest and contributions of fathers to child development and the potential benefits to mother and child when fathers are engaged in early phases of parenthood. Early intervention efforts provide opportunities for young fathers to build their capacities to serve as protective (rather than negative) influences on child development (Kotila et al., 2015). While the development of programs for young fathers has continued to grow, there is still a need for more rigorous evaluation research that includes mixed methods and follow‐up studies to better understand long‐term impacts on family and child outcomes. As family structures and parental roles continue to evolve, it will be important to support all parents (to the extent possible) in reproductive decision‐making and parenting practice that ensure healthy child development and provide a foundation for nurturing next‐generation mothers and fathers (Parke, 2013a).

## APPENDIX A

### Case illustration

**Table:**

Seventeen‐year‐old Jessica was 6 months pregnant as a senior in high school. Jamal, the 19‐year‐old father, graduated from the same high school, worked part‐time, and attended the local community college. They had been together for about a year and were both excited and anxious about becoming parents. Jamal lived with his cousin, and Jessica lived with her single mother and younger brother. Jessica and Jamal had talked about living together after the birth of the baby, but Jessica's mother was not supportive of this, and it was difficult financially to afford an independent apartment.
Jessica was referred by her healthcare provider to Early Head Start for prenatal home visits, and she enrolled in her school program for teen mothers. James attended the first ultrasound, but was not included in the prenatal home visits and was not able to attend other prenatal health visits due to his work schedule.
Jamal hoped to be present at the birth of his child, but was excluded on the advice of Jessica's mother. However, he was happy to see and hold the baby a few days after the birth. He occasionally visited his son and brought presents during the first few months, but did not feel welcome and began visiting less often. Jamal struggled to get a better job and soon dropped out of school to work full‐time to support his child.
In time, Jessica started college as a single parent at a local state university. Her mother helped with childcare, and she received tuition and housing assistance through special programs for single parents. She also enrolled in a parenting support program. Occupied with work, Jamal visited less frequently and soon became involved in a new adult relationship. As a 4‐year‐old, struggling in childcare, their son wondered why his father no longer visited.

## APPENDIX B

### Core principles for ethical relational discourse

**Table:**

Core Principles for Ethical Relational Discourse
Adapted from *Ethical Thinking and Practice for Parent and Family Life Educators*
Minnesota Council on Family Relations, 2016
**I**. **Relationships with Parents** Maintain awareness of impact providers have on parents and families. Respect cultural beliefs, backgrounds, and differences and engage in practice that is sensitive to the diversity of child‐rearing values and goals. Help parents and family members recognize and work with strengths to set goals. Communicate respectfully and clearly with all family members. Include parents and family members as partners in problem‐solving and decision‐making. Provide a program environment that is safe and nurturing to all family members.
**II**. **Relationships with Mothers** Respect a mother's beliefs and motivation about becoming a parent. Support a mother's efforts to care for herself and her child during pregnancy. Respect a mother's rights and control over reproduction. Respect and protect a mother's right to be safe from Intimate Partner Violence Support a mother in building their capacity as parents and co‐parents.

**Table:**

Core Principles for Ethical Relational Discourse
Adapted from *Ethical Thinking and Practice for Parent and Family Life Educators*
Minnesota Council on Family Relations, 2016
**III**. **Relationships with Fathers** Recognize fathers as important to children and can be an under‐utilized resource. Respect fathers’ beliefs and motivations about becoming a parent. Acknowledge that fathers care deeply about their biological children. Support fathers in building their capacity as parents. Support fathers and their role to be a parenting partner with mothers.
**IV**. **Relationships with Children (Prenatal to Three)** Treat children with respect and sensitivity to their needs and rights as developing persons. Strive to understand children in the context of their families Do no harm to children and insist the same from others. Advocate for children and their best interests while working with parents. Support the right of all children to have access to quality education, health, and community resources.
